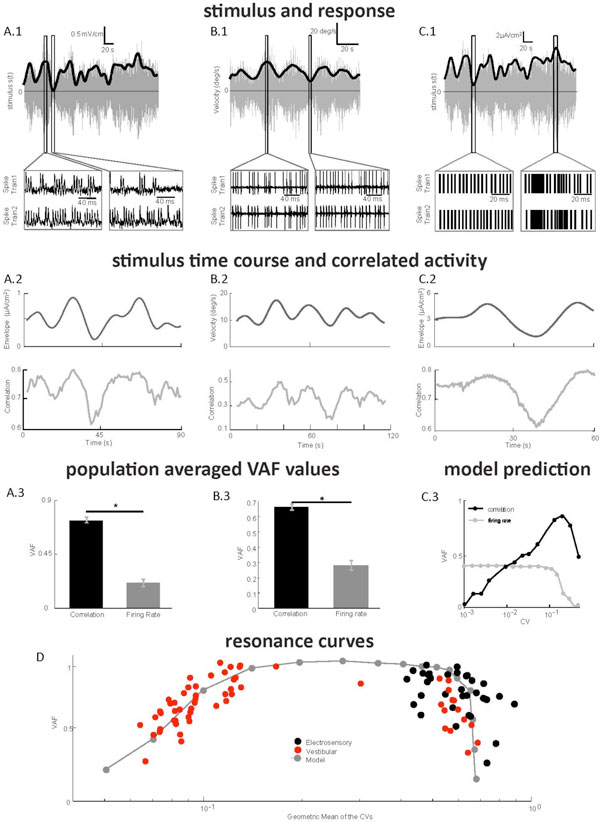# Neural correlations code for stimulus variance

**DOI:** 10.1186/1471-2202-14-S1-P61

**Published:** 2013-07-08

**Authors:** Michael G Metzen, Mohsen Jamali, Jerome Carriot, Oscar Avila-Akerberg, Kathleen E Cullen, Maurice J Chacron

**Affiliations:** 1 Physiology, McGill University, Montreal, Quebec, Canada, H3G1Y6

## 

Natural sensory stimuli are characterized by time varying moments such as mean (first-order) and variance (second-order). While psychophysical studies have shown that second order attributes are critical for perception, how they are encoded in the brain remains largely unknown. Here we focused on second-order feature coding by correlated activity. We recorded from two example sensory systems that share many similarities: the primate vestibular system and the fish electrosensory system. Peripheral sensory neurons in both systems respond to amplitude modulated noise stimuli (Figure. 1A.1, B.1). We found that the correlation coefficient between spike trains coded second order attributes (i.e. envelope, Figures. 1A.2, B.2) whereas single neuron firing rate coded first order attributes (Figure. 1A.3, B.3). We built a simple phenomenological mathematical model based on the leaky integrate-and-fire formalism that reproduced our experimental data (Figure. 1C.1, C.2) and predicted that optimal coding of second-order stimulus features by correlation is achieved for non-zero values of baseline variability as quantified by the coefficient of variation (CV) (Figure. 1C.3). We tested this prediction by plotting our data as a function of CV and found that our model could explain variations on both vestibular and electrosensory data (Figure. 1D). Our results show that correlated activity codes for stimulus attributes that are distinct from those coded by firing rate and provide a novel role for neural variability. Such codes are predicted to be a general feature of sensory processing.

**Figure 1:**